# Adjacent-room dual-console Remote Surgical Training (ReST) with takeover capability on the da Vinci Xi: a porcine-model feasibility study

**DOI:** 10.1007/s11701-026-03391-9

**Published:** 2026-04-21

**Authors:** Lorenzo Spirito, Carmine Sciorio, Vittorio Imperatore, Antonio Di Girolamo, Giuseppe Romeo, Riccardo Giannella, Antonio Ruffo, Fabio Esposito, Lorenzo Romano, Paola Coppola, Luigi Napolitano, Antonio D’Ambrosio, Roberta Siciliano, Guido De Sena

**Affiliations:** 1 Department of Woman Child General and Specialized Surgery, University of Campania “Luigi Vanvitelli”, Caserta, Italy; 2 Alessandro Manzoni Hospital, Lecco, Italy; 3 Azienda Ospedaliera S.Giuseppe Moscati, Avellino, Italy; 4 Ospedale Antonio Cardarelli, Naples, Italy; 5 Casa di Cura Nostra signora di Lourdes, Naples, Italy; 6 Department of Economics and Statistics, University of Naples Federico II, Naples, Italy; 7 Dipartimento di Ingegneria Elettrica e dell’Informazione, University of Naples Federico II, Naples, Italy; 8 Department of General Surgery, Ospedale Antonio Cardarelli, Naples, Italy

**Keywords:** Robotic surgery education, Dual-console training, Adjacent-room remote surgical training, GEARS, Porcine model, Robotic mentoring

## Abstract

We evaluated a Remote Surgical Training model (ReST) in which the tutor operates a second da Vinci Xi console from an adjacent room while preserving integrated audio and the ability to take over control. A comparative feasibility study was conducted at RAIN (A. Cardarelli Hospital, Naples, Italy) between March 2021 and April 2023 using a porcine model. Thirty training sessions were analysed (15 ReST, 15 in-room dual-console controls). Trainee performance was assessed with the Global Evaluative Assessment of Robotic Skills (GEARS); post-session surveys captured perceptions, tutor-trainee agreement (weighted Cohen’s κ), priority rankings (Kemeny aggregation with τX) and satisfaction. GEARS internal consistency was acceptable (Cronbach’s α overall 0.805; control 0.837; ReST 0.768). No statistically significant differences were observed for the GEARS total score (Mann-Whitney U = 105, p = 0.595) or domains (p = 0.074–0.683). Tutor-trainee agreement was significant for three interaction items: ease of teaching/training (p = 0.004), ability to follow the trainee’s actions (p = 0.011) and overall interaction quality (p = 0.014). Priority rankings in both roles emphasised clarity of instruction and takeover capability, while physical proximity ranked lowest. Tutors and trainers reported high satisfaction in both groups; one trainee reported low satisfaction in the ReST group. Adjacent-room ReST with takeover capability was feasible in this porcine-model training setting and showed no observed performance decrement compared with conventional in-room dual-console training. These findings should not be interpreted as proof of equivalence or as validation of true long-distance teleproctoring; further work is required for larger samples, participant-level baseline adjustment, and longer-distance configurations.

**Supplementary Information:**

The online version contains supplementary material available at 10.1007/s11701-026-03391-9.

## Introduction

Robot-assisted surgery continues to expand across specialties and procedure types, while the technical and cognitive demands of console-based surgery remain substantial. Mastery of robotic techniques is characterised by learning curves that vary by procedure and by the metrics used to define proficiency. Systematic evidence indicates that a considerable number of cases may be required to achieve stable performance across a range of robotic operations, underscoring the importance of structured training and supervised progression [1].

Modern robotic curricula increasingly combine simulator-based practice, modular task training and supervised clinical exposure. These approaches aim to accelerate skill acquisition while maintaining patient safety and optimising the use of operating room resources. Nevertheless, at the point of transition from simulation to live tissue or clinical procedures, trainees still rely on high-quality real-time guidance, feedback and, when necessary, direct intervention by experienced robotic surgeons [2].

Dual-console platforms have become a cornerstone for intraoperative mentoring. They permit real-time verbal instruction, shared visual context, pointer-based guidance and console takeover, thereby supporting progressive autonomy while preserving the tutor’s ability to intervene when needed [3–6]. Video-based analysis has shown that the structure of teaching interactions and the timing of console switching can influence learning opportunities and operative flow [6]. In addition, observational analyses suggest that the presence of a trainee at the console may influence operative metrics, reinforcing the need for supervision models that are both educationally effective and operationally acceptable [7]. Dual-console training is widely used to balance autonomy and safety during the learning curve. The COVID-19 pandemic substantially disrupted access to operative training, motivating the development of resilient training frameworks and alternative mentorship models [8].

Despite these advantages, conventional dual-console training requires the physical co-location of tutor and trainee in the operating room. Co-location can be constrained by organisational factors (availability of expert mentors, competing clinical duties), infection-prevention policies and limitations in access to specialised training centres. These constraints have stimulated interest in teleproctoring and telementoring approaches intended to extend expert guidance beyond the physical operating room [9].

Remote proctoring can offer visual guidance, structured feedback and decision support; however, many telementoring workflows are designed primarily for observation and advice and may not permit direct hands-on intervention. In robotic surgery, the ability to take over control remains a particularly relevant safety feature and differentiates dual-console training from many teleproctoring configurations [10]. The field’s trajectory toward remote surgery is well illustrated by early telesurgery milestones, which demonstrated feasibility but also emphasised technical, regulatory and governance challenges [11].

From a technical perspective, remote surgical workflows are sensitive to network quality, including bandwidth, stability and latency, which can affect video transmission and command execution. Experimental work has shown that bandwidth limitation can influence remote surgical performance, supporting the need for systematic assessment of connectivity when moving beyond local configurations [12]. Recent demonstrations of long-distance robotic procedures using advanced connectivity, including fifth-generation networks, further underscore the potential for distributed models of surgical support while reinforcing the need for rigorous safety and quality frameworks [13].

An intermediate configuration that preserves key safety and educational features while reducing the requirement for full in-room co-location may therefore be valuable. We evaluated a Remote Surgical Training model (ReST) in which the tutor console is placed in an adjacent room within the same operating block, maintaining integrated audio communication and preserving the possibility of tutor takeover. We assessed performance using the Global Evaluative Assessment of Robotic Skills (GEARS), a validated tool with external validation for the structured evaluation of robotic surgical skills across multiple domains [14, 15]. In parallel, multi-actor perception and prioritisation data were collected to capture human factors elements that shape mentoring effectiveness and acceptability.

The primary aim was to assess the feasibility of implementing adjacent-room ReST on the da Vinci Xi system in an animal-model setting and to explore whether any observable differences emerged in trainee performance compared with conventional in-room dual-console sessions. Secondary aims were to characterise tutor-trainee agreement on perceived interaction quality, identify priority factors for effective training through rank aggregation, and describe satisfaction across participant roles.

## Materials and methods

### Study design and setting

This was a comparative feasibility study conducted at the Robotic Academy Intuitive Naples (RAIN), A. Cardarelli Hospital (Naples, Italy). Training activities were performed using a da Vinci Xi robotic platform (Intuitive Surgical, Sunnyvale, CA, USA) in a dual-console configuration within a dedicated robotic operating room. The overall study period spanned March 2021 to April 2023. All sessions were performed on a porcine model within the institutional training environment, within the scope of institutional robotic training activities (including general urological, gynecological, and thoracic surgery).

A total of 30 training sessions were included in the comparative analysis: 15 ReST tests and 15 conventional in-room dual-console control sessions. The unit of analysis was the individual training event/session, defined by a tutor-trainee pairing conducted within one operating room session and followed by structured assessment (GEARS) and post-session surveys. Each session therefore contributed one set of performance and perception measurements to the dataset. The group size was pragmatic, reflecting sessions accrued during the institutional training programme; no formal sample size calculation, randomisation, or matching procedures were reported. Missing data were not imputed; analyses were performed on complete observations for each outcome (available-case analysis), with denominators to be reported where applicable.

### Participants and roles

Each session involved a trainee, a tutor and a specialist trainer/supervisor. For the purposes of this study, the tutor was defined as the experienced robotic surgeon responsible for real-time instruction, supervision and, when required, direct demonstration through console takeover. The trainee was the surgeon in training who performed the console tasks and procedural steps on the porcine model. A specialist trainer/supervisor was physically present in the operating room to oversee the training workflow and safety, to provide additional guidance when needed, and to support performance assessment. In this manuscript, the term experienced surgeon refers to the tutor and/or the in-room specialist trainer/supervisor, depending on the role being described. Trainees comprised senior residents, fellows, and early-career specialists with prior bedside-assistant exposure and limited supervised robotic console experience.

Post-session surveys were completed by the tutor, trainee and specialist trainer/supervisor at the end of each training event using a real-time data-collection platform prepared for the project. Performance assessment using the GEARS tool was completed by supervisors present in the operating room in accordance with the institutional training workflow. Because the session was the unit of analysis, session-level baseline descriptors are reported to contextualise the findings; these data are intended to describe the pragmatic training cohort rather than to imply formal matching. Table 1 reports session-level rather than unique participant-level characteristics; because repeated participation by the same trainee or tutor could not be modelled in the present dataset, these descriptors should be interpreted as contextual rather than matched baseline data.

**Table 1:** Session-level characteristics by study group

Characteristic	Control	ReST
Sessions analysed, n	15	15
Specialty distribution, n sessions	Urology 8; Gynecology 4; Thoracic 3	Urology 8; Gynecology 4; Thoracic 3
Trainee stage, n sessions	Senior resident 7; Fellow 5; Junior attending 3	Senior resident 6; Fellow 6; Junior attending 3
Prior robotic console cases, median [IQR]	8 [4–12]	10 [5–15]
Prior bedside-assistant robotic cases, median [IQR]	15 [10–25]	18 [11–26]
Tutor years of robotic practice, median [IQR]	7 [5–10]	7 [5–9]
Specialist trainer/supervisor present, n (%)	15 (100%)	15 (100%)

### Intervention: ReST vs. control

Control condition (in-room dual console): In the control sessions, the tutor and trainee consoles were both located in the same robotic operating room. The tutor provided real-time instruction via direct communication and shared situational awareness and could take over control of the robotic system to demonstrate specific operative steps or to manage a training-critical moment. The overall training workflow mirrored the institutional protocol used at RAIN (TR300-like structure), with supervision by the specialist trainer/supervisor in the operating room.

ReST condition (adjacent-room dual console): In the ReST sessions, the same dual-console training workflow was maintained, with the key difference that the tutor console was positioned in an adjacent room within the same operating block. The tutor console was connected to the da Vinci Xi system through a long cable, and bidirectional audio communication was maintained using the integrated da Vinci Xi audio system. To support situational awareness, an environmental camera within the operating room could be remotely controlled by the tutor. Crucially, the tutor retained the ability to take over control of the robotic system and instruments for demonstration or intervention, preserving a central safety feature of dual-console training while introducing physical separation from the operating room.

Depending on the specialty-specific session, the trainee-performed console phase comprised predefined operative modules including tissue exposure, blunt/sharp dissection, atraumatic tissue handling, vessel sealing or clipping, limited intracorporeal suturing/knot tying, and selected procedural steps representative of the target specialty. GEARS scoring was restricted to the active trainee console phase and did not include animal preparation, anaesthetic preparation, docking, or non-console bedside tasks.

Each session followed a structured workflow consisting of pre-session briefing, console allocation/orientation, live console training with integrated audio communication, tutor takeover when required, immediate GEARS completion by the in-room supervisor, and post-session debriefing plus questionnaire completion. In ReST sessions, the remote tutor also had access to the environmental camera to support situational awareness from the adjacent room. The same institutional dual-console training framework was used in both groups; the between-group difference concerned tutor location and the resulting physical separation, rather than a different curricular structure.

### Outcomes and assessment

Performance outcome (GEARS): Trainee performance during each session was evaluated using the Global Evaluative Assessment of Robotic Skills (GEARS) tool. GEARS comprises seven domains: depth perception, bimanual dexterity, efficiency, force sensitivity, autonomy, robotic control and use of the third arm; each is scored on an ordinal scale. A total GEARS score was calculated as the average across domains.

Perceptions, priorities and satisfaction: Immediately after each session, structured surveys captured perceptions of the training interaction from tutors, trainees and specialist trainers/supervisors. Survey responses used a four-level Likert format (strongly agree, agree, disagree, strongly disagree). A subset of items was shared between tutors and trainees to enable direct assessment of agreement (e.g., ease of teaching/training, ability of the tutor to follow the trainee, interaction quality, clarity and promptness of instructions). Participants also completed a ranking task to prioritise factors considered most important for an effective training session. Overall satisfaction with the session was recorded using an ordinal satisfaction scale and summarised graphically. The tutor, trainee, and specialist trainer/supervisor questionnaires, together with the supplementary item-level perception tables and satisfaction counts, are provided in Online Resource 1. Descriptive field notes were also recorded during selected sessions to document operational features such as remote use of the environmental camera; these notes were not subjected to formal qualitative analysis.

### Statistical analysis

Internal consistency and group comparisons: Internal consistency of GEARS was assessed using Cronbach’s alpha overall and within each study group. Group summaries are reported as medians [interquartile range]. Between-group comparisons (ReST vs. control) were performed using the Mann-Whitney U test for the total GEARS score and for each GEARS domain, as per the original analysis plan. In addition to p-values, effect sizes for Mann-Whitney comparisons are reported as the absolute rank-biserial correlation (|r_rb|) derived from the U statistic and group sample sizes (n1 = n2=15).

Agreement, ranking and similarity analyses: Tutor-trainee agreement on shared survey items was evaluated using weighted Cohen’s kappa; p-values are reported for the hypothesis of agreement beyond chance. Priority rankings were aggregated using an axiomatic Kemeny approach to derive a consensus ordering for each role and group, and within-sample agreement was quantified using the averaged τX rank correlation coefficient (reported for the whole sample and by group). To explore whether response patterns within the same actor category were similar between control and ReST sessions, Spearman correlation coefficients were calculated across items, with the observed range reported. Analyses were exploratory in nature; no additional post hoc analyses were introduced.

### Ethics and animal welfare

The study was conducted on a porcine model within an institutional robotic training environment at RAIN (A. Cardarelli Hospital, Naples, Italy). Procedures were performed on pigs as part of training activities under authorization no. DGSAF/2021/REST-XI/017, approved by the competent institutional and national animal-care body. Animals were managed according to applicable institutional and national standards, with general anaesthesia, perioperative analgesia, and predefined humane endpoints.

## Results

### Study sessions overview

Thirty training sessions were included in the analysis: 15 ReST tests and 15 in-room dual-console control sessions, all conducted on a porcine model within the RAIN training environment. For each training event, a GEARS assessment and the post-session surveys (tutor, trainee and specialist trainer/supervisor) were completed at the end of the session as part of the study workflow.

### GEARS: reliability and between-group comparison

GEARS reliability: GEARS demonstrated acceptable internal consistency in this dataset, with Cronbach’s alpha of 0.805 overall. When calculated within groups, Cronbach’s alpha was 0.837 for control sessions and 0.768 for ReST sessions.

Between-group comparison: No statistically significant differences were observed between ReST and control for the total GEARS score (Mann-Whitney U = 105, *p* = 0.595). Group summaries and domain-level comparisons are summarised in Table 2. Across the seven GEARS domains, p-values ranged from 0.074 (robotic control) to 0.683 (use of the third arm), and no domain reached statistical significance. Effect size estimates based on the absolute rank-biserial correlation (|r_rb|) were small overall (range 0.093–0.387), with the largest magnitude observed for robotic control (|r_rb| = 0.387).

**Table 2:** GEARS descriptive statistics and Mann-Whitney U comparison between control and ReST sessions

GEARS domain	Control median [IQR]	ReST median [IQR]	U	*p*	Abs. rank-biserial *r*
Depth perception	3.7 [3.4–4.0]	3.6 [3.3–4.0]	96	0.512	0.147
Bimanual dexterity	3.8 [3.5–4.1]	3.8 [3.4–4.0]	124	0.653	0.102
Efficiency	3.6 [3.3–3.9]	3.5 [3.2–3.8]	84	0.250	0.253
Force sensitivity	3.5 [3.2–3.9]	3.4 [3.1–3.8]	97	0.539	0.138
Autonomy	3.4 [3.1–3.8]	3.7 [3.4–4.0]	151	0.106	0.342
Robotic control	3.8 [3.5–4.1]	3.4 [3.1–3.8]	69	0.074	0.387
Use of third arm	3.4 [3.1–3.7]	3.5 [3.1–3.8]	102	0.683	0.093
Total GEARS score	3.60 [3.39–3.84]	3.57 [3.32–3.81]	105	0.595	0.067

Abs. rank-biserial r = absolute rank-biserial correlation

### Perception survey: agreement and trainer perspective

Tutor-trainee agreement: Across the 30 tutor-trainee paired sessions, significant agreement was observed for the item pair addressing whether the session made teaching/training easy (tutors: 12/30 strongly agree, 15/30 agree, 3/30 disagree; trainees: 10/30 strongly agree, 17/30 agree, 3/30 disagree; weighted κ = 0.47, *p* = 0.004), the ability of the tutor to follow the trainee’s actions / the trainee’s feeling of being adequately followed (tutors: 11/30 strongly agree, 16/30 agree, 3/30 disagree; trainees: 9/30 strongly agree, 17/30 agree, 4/30 disagree; weighted κ = 0.42, *p* = 0.011), and overall interaction quality (tutors: 13/30 strongly agree, 15/30 agree, 2/30 disagree; trainees: 12/30 strongly agree, 15/30 agree, 3/30 disagree; weighted κ = 0.39, *p* = 0.014).

Across the remaining shared items, promptness/clarity of instruction showed uniformly positive responses (tutors: 17/30 agree, 13/30 strongly agree; trainees: 12/30 agree, 18/30 strongly agree; weighted κ = 0.18, *p* = 0.170). For achievement of learning objectives, tutors responded disagree/agree/strongly agree in 2/30, 18/30, and 10/30 sessions and trainees in 3/30, 19/30, and 8/30 sessions (weighted κ = 0.13, *p* = 0.290). Perceptions of physical closeness were more discordant: tutors responded strongly disagree/disagree/agree/strongly agree in 16/30, 10/30, 3/30, and 1/30 sessions, whereas trainees responded 5/30, 9/30, 10/30, and 6/30 sessions, respectively (weighted κ = 0.05, *p* = 0.680). Full item-level counts, weighted κ values, and p-values are provided in Online Resource 1, Table S1.

Specialist trainer/supervisor perspective and between-group similarity: Among specialist trainers/supervisors, learning atmosphere was rated agree/strongly agree in all sessions (control: 5/15 agree and 10/15 strongly agree; ReST: 4/15 agree and 11/15 strongly agree). Interaction quality between tutor and trainee was rated disagree/agree/strongly agree in 1/15, 5/15, and 9/15 control sessions and in 0/15, 3/15, and 12/15 ReST sessions. Perceived safe replicability on real patients was rated disagree/agree/strongly agree in 1/15, 5/15, and 9/15 control sessions and in 1/15, 4/15, and 10/15 ReST sessions. When comparing the pattern of item responses within the same actor category between control and ReST sessions, Spearman correlation coefficients ranged from − 0.4 to 1.0 across the surveyed items, indicating heterogeneous similarity depending on the construct assessed. Trainer item distributions are provided in Online Resource 1, Table S2.

### Priorities ranking

Consensus priority rankings derived using the Kemeny approach are presented for tutors (Table 3) and trainees (Table 4). Across both roles and both conditions, priorities consistently placed clarity of instruction and the ability for the tutor to intervene/take over among the highest-ranked factors, whereas physical proximity between tutor and trainee was ranked lowest.

**Table 3:** Tutor priority rankings and averaged τX rank correlation coefficient

Priority item	Whole sample (rank)	Control (rank)	ReST (rank)
The trainee follows my instructions carefully	1	1	1
I can take over the controls	2	3	2
I have a feeling with the trainee	3	2	3
I feel comfortable during interaction	4	4	4
The trainee stands close to me	5	5	5

Averaged τX rank correlation: whole sample 0.5133; control 0.3867; ReST 0.6800

**Table 4:** Trainee priority rankings and averaged τX rank correlation coefficient

Priority item	Whole sample (rank)	Control (rank)	ReST (rank)
The instructions given by the tutor are clear	1	1	1
The tutor can follow my actions	2	2	2
I feel comfortable and autonomous during the interaction	3	4	3
The tutor can take over the controls	4	3	4
The tutor stands close to me	5	5	5

Averaged τX rank correlation: whole sample 0.3933; control 0.3733; ReST 0.4267

Tutors: In the whole sample, the highest-ranked tutor priority was that the trainee follows instructions carefully, followed by the ability of the tutor to take over control. In the control group, the importance of “having a feeling with the trainee” increased relative to the ReST group, consistent with the greater emphasis on in-room co-presence. Agreement across tutors, quantified by τX, was moderate overall (0.5133) and differed by group (control 0.3867; ReST 0.6800).

Trainees: In the whole sample, trainees ranked clarity of instruction as the top priority and the perception that the tutor can follow their actions as second. Comfort/autonomy during the session and the ability of the tutor to take over were ranked in the middle positions, with physical proximity consistently ranked last. Agreement across trainees was lower than across tutors (τX overall 0.3933) but was similar between groups (control 0.3733; ReST 0.4267).

### Satisfaction

**Fig. 1:**
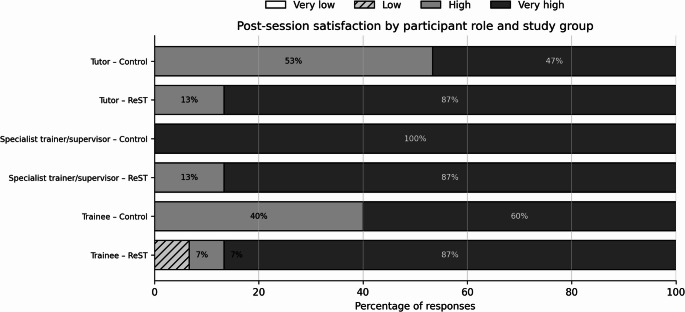
Distribution of post-session satisfaction responses (very low, low, high, very high) by participant role and study group; grayscale rendering used for print-safe readability

Tutor satisfaction was high or very high in all sessions (control: 8/15 high and 7/15 very high; ReST: 2/15 high and 13/15 very high). Specialist trainer/supervisor satisfaction was high or very high in all sessions (control: 15/15 very high; ReST: 2/15 high and 13/15 very high). Trainee satisfaction was predominantly high or very high (control: 6/15 high and 9/15 very high; ReST: 1/15 low, 1/15 high, and 13/15 very high), with one low trainee rating in the ReST group (Fig. 1; Online Resource 1, Table S3).

### External observation

External observation notes recorded during the study indicated that, in ReST sessions, the remote tutor actively used the environmental camera to monitor the operating room environment and to support situational awareness while providing guidance from the adjacent room.

## Discussion

Robotic surgery training increasingly depends on structured mentorship, yet expert co-location can be constrained by organisational factors and infection-control policies. In this comparative feasibility study of 30 porcine-model dual-console sessions (15 adjacent-room ReST, 15 in-room controls), three findings stand out: (i) the ReST setup was operationally feasible while preserving integrated audio communication, environmental-camera situational awareness and immediate tutor takeover; (ii) no statistically significant differences were observed in trainee performance on GEARS (total *p* = 0.595; domain *p* = 0.074–0.683); and (iii) perception and ranking data consistently prioritised clarity of instruction and takeover capability over physical proximity.

Performance findings should be interpreted conservatively. The absence of statistically significant differences does not demonstrate equivalence or non-inferiority; rather, under the specific conditions tested (single centre, adjacent room, long-cable connection, animal model), no clear performance decrement was detected. GEARS showed acceptable internal consistency overall (Cronbach’s α 0.805) and within groups (0.837 control; 0.768 ReST), supporting its use as a structured metric in this context [14, 15]. The borderline signal for the “robotic control” domain (*p* = 0.074) may reflect limited power with *n* = 15 per group rather than a true difference, but it remains a useful focus for future confirmatory designs. Importantly, adjacent-room ReST is a “local remote” configuration that minimises network-related uncertainty compared with true long-distance mentoring; as remote workflows move beyond local wired connections, bandwidth and latency become increasingly influential on surgeon experience and performance [12, 13].

The human factors results clarify why an adjacent-room model can remain educationally functional despite physical separation. Tutor-trainee agreement reached statistical significance for three interaction items (ease of teaching/training, ability to follow the trainee’s actions, and overall interaction quality), suggesting that the core teaching interface can remain aligned when audio, endoscopic view sharing and takeover are preserved. In parallel, both tutors and trainees ranked physical proximity as the lowest priority, whereas clarity of instruction and takeover capability occupied the top positions. These priorities are coherent with what has been observed in in-room dual-console teaching: the educational value often hinges on timely coaching, shared situational awareness and effective console switching rather than mere co-presence [6]. The environmental camera—actively used by the remote tutor in observational notes—may have contributed to situational awareness by restoring some of the non-console cues (team positioning, room activity) that would otherwise be lost. Satisfaction was high for tutors and trainers in both groups, with one low trainee satisfaction response in the ReST arm, indicating that acceptability may vary across individuals even when performance metrics do not diverge.

Within the broader landscape of remote guidance, adjacent-room ReST can be considered an intermediate configuration between conventional in-room dual-console mentoring and teleproctoring/telementoring. Systematic reviews of robotic teleproctoring report feasibility across platforms but emphasise heterogeneity in technical setups, outcomes and governance, and many configurations rely primarily on observation and verbal guidance without the safety affordance of direct takeover [16]. Educational syntheses of surgical telementoring similarly highlight that the effectiveness of remote supervision depends on deliberate instructional design, reliable audiovisual interaction and a clearly defined escalation pathway for patient safety [17]. In contrast to long-distance telesurgery paradigms—historically exemplified by transcontinental demonstrations [11] and more recent connectivity-enabled concepts [13]—the adjacent-room model keeps the technical risk envelope relatively narrow while still probing the key human factors question: what is lost, and what can be compensated for, when physical proximity is removed but interactivity and takeover remain? From a governance perspective, best-practice recommendations for telesurgery stress credentialing, responsibility allocation, cybersecurity and contingency planning [18]; these considerations become mandatory as programmes progress from “adjacent room” to inter-hospital or cross-jurisdiction configurations.

From an implementation standpoint, the ranking findings are particularly actionable: they suggest that, for many participants, the perceived quality of training is driven more by communication clarity and the ability to intervene than by the tutor’s physical presence. For adjacent-room ReST, this implies that standardising verbal instruction (closed-loop communication, explicit step labels), formalising takeover criteria, and ensuring that environmental awareness tools (e.g., controllable room camera) are reliable may yield more benefit than attempting to recreate in-room proximity. At the same time, the divergence in perceptions about the need for physical closeness indicates that programmes should not assume a uniform trainee preference; structured pre-briefing about the remote setup and explicit expectations about when takeover will occur may help mitigate uncertainty and improve acceptability.

Several limitations warrant caution. First, this was an animal-model feasibility study; generalisability to patient surgery and to higher-stakes clinical contexts is uncertain. Second, “remote” here meant an adjacent-room configuration within the same operating block; the study does not address network latency, bandwidth instability, cross-institution credentialing, licensure, cybersecurity, or liability frameworks that define long-distance teleproctoring or telesurgery. Third, the sample was pragmatic and limited (15 sessions per group), analysed at the session level, and not based on a formal sample-size calculation, randomisation, or matching. Fourth, trainee seniority, prior robotic exposure, specialty-specific session content, and the constant presence of an in-room specialist trainer/supervisor may have influenced both GEARS and perception outcomes. Because repeated participation by the same trainees or tutors could not be modelled, residual confounding cannot be excluded and no non-inferiority or equivalence claim can be made. Accordingly, the present findings should not be extrapolated to bandwidth-dependent, latency-sensitive, cross-site teleproctoring or telesurgery configurations. Future studies should include larger samples, participant-level baseline descriptors, fuller agreement reporting, and designs explicitly tailored to the intended claim, alongside pre-specified governance procedures before extending beyond local configurations [18].

## Conclusions

Adjacent-room Remote Surgical Training (ReST) on the da Vinci Xi platform was feasible in this porcine-model setting and did not show an observed performance decrement compared with in-room dual-console training. These findings should not be interpreted as proof of equivalence or as validation of true long-distance teleproctoring or telesurgery. Further prospective work should include larger samples, participant-level baseline descriptors, and longer-distance network configurations before clinical generalization is considered.

## Supplementary Information

Below is the link to the electronic supplementary material.

Supplementary Material 1

## Data Availability

De-identified data supporting the findings of this study (GEARS domain scores and survey responses) are available from the corresponding author upon reasonable request and subject to institutional approvals and data-use conditions.

## Declarations

### Use of AI tools

An AI language model was used to support language editing during manuscript preparation. All authors reviewed and take responsibility for the content.

### Supplementary information

Online Resource 1 provides the tutor, trainee, and specialist trainer/supervisor questionnaires, item-level perception tables, satisfaction counts, and the revised satisfaction figure.
