# Supplementary material for: Adjacent-room dual-console Remote Surgical Training (ReST) with takeover capability on the da Vinci Xi: a porcine-model feasibility study
Source: J Robot Surg. 2026 Apr 21;20(1):438. doi: 10.1007/s11701-026-03391-9 (PMC13095934; doi:10.1007/s11701-026-03391-9)
Supplement: Supplementary file 1 — Supplementary Material 1 [file 11701_2026_3391_MOESM1_ESM.pdf]

Online Resource 1

Adjacent-Room Dual-Console Remote Surgical Training (ReST) with Takeover Capability on the da Vinci Xi: A Porcine-Model Feasibility Study

Journal of Robotic Surgery

Lorenzo Spirito, Carmine Sciorio, Vittorio Imperatore, Antonio Di Girolamo, Giuseppe Romeo, Riccardo Giannella, Antonio Ruffo, Fabio Esposito, Lorenzo Romano, Paola Coppola, Luigi Napolitano, Antonio D'Ambrosio, Roberta Siciliano, Guido De Sena

This file contains the tutor, trainee, and specialist trainer/supervisor post-session questionnaires; item-level perception tables; satisfaction counts; and a grayscale-safe redraw of the satisfaction figure.

Correspondence to: Lorenzo Spirito, MD, PhD

**Article title**	**Adjacent-Room Dual-Console Remote Surgical Training (ReST) with Takeover Capability on the da Vinci Xi: A Porcine-Model Feasibility Study**
Supplement caption	Online Resource 1. Tutor, trainee, and specialist trainer/supervisor post-session questionnaires; item-level perception tables; satisfaction counts; revised satisfaction figure.

Questionnaire 1 - Tutor

Post-session form completed by the tutor.

**Session ID:** ____________________	**Date:** ____________________	**Specialty:** ____________________
**Group (Control / ReST):** ____________________	**Tutor code:** ____________________	**Trainee code:** ____________________
**Trainer code:** ____________________		

Section A. Perception items (4-point Likert scale)

No.	Statement	Strongly disagree	Disagree	Agree	Strongly agree
1	The session made surgical teaching easy.	☐	☐	☐	☐
2	I could adequately follow the trainee during the console tasks.	☐	☐	☐	☐
3	The quality of interaction with the trainee was good.	☐	☐	☐	☐
4	The trainee applied my instructions promptly.	☐	☐	☐	☐
5	My function was effective in achieving the trainee's learning objectives.	☐	☐	☐	☐
6	I felt comfortable during the interaction.	☐	☐	☐	☐
7	I felt the need to have the trainee physically close by.	☐	☐	☐	☐
8	I could take over the commands at the right moment when needed.	☐	☐	☐	☐
9	This format could be replicated safely on real patients.	☐	☐	☐	☐

Section B. Priority ranking (1 = most important; 5 = least important)

Item	Rank (1-5)
The trainee can follow my instructions carefully	_____
I can take over the commands at the right moment	_____
I have feeling with the trainee	_____
I feel comfortable	_____
The trainee stands close to me	_____

Section C. Overall satisfaction

The overall satisfaction of my experience with surgical tutoring was:

Very low ☐ Low ☐ High ☐ Very high ☐

Questionnaire 2 - Trainee

Post-session form completed by the trainee.

**Session ID:** ____________________	**Date:** ____________________	**Specialty:** ____________________
**Group (Control / ReST):** ____________________	**Trainee code:** ____________________	**Tutor code:** ____________________
**Trainer code:** ____________________		

Section A. Perception items (4-point Likert scale)

No.	Statement	Strongly disagree	Disagree	Agree	Strongly agree
1	The session made surgical training easy.	☐	☐	☐	☐
2	I felt adequately followed by the tutor in my tasks.	☐	☐	☐	☐
3	The quality of interaction with the tutor was good.	☐	☐	☐	☐
4	The instructions given by the tutor were clear and understandable.	☐	☐	☐	☐
5	This session was effective in achieving my learning objectives.	☐	☐	☐	☐
6	I felt comfortable and autonomous during the interaction.	☐	☐	☐	☐
7	I felt the need to have the tutor physically close by.	☐	☐	☐	☐
8	The tutor took over the commands at the right moment when needed.	☐	☐	☐	☐
9	This format could be replicated safely on real patients.	☐	☐	☐	☐

Section B. Priority ranking (1 = most important; 5 = least important)

Item	Rank (1-5)
The instructions of the tutor are clear and understandable	_____
The tutor follows me in my tasks	_____
I feel comfortable and autonomous	_____
The tutor takes over the commands at the right moment	_____
The tutor stands close to me	_____

Section C. Overall satisfaction

The overall satisfaction of my experience with surgical training was:

Very low ☐ Low ☐ High ☐ Very high ☐

Questionnaire 3 - Specialist trainer / supervisor

Post-session form completed by the in-room specialist trainer/supervisor.

**Session ID:** ____________________	**Date:** ____________________	**Specialty:** ____________________
**Group (Control / ReST):** ____________________	**Trainer code:** ____________________	

Section A. Perception items (4-point Likert scale)

No.	Statement	Strongly disagree	Disagree	Agree	Strongly agree
1	The atmosphere for learning was adequate.	☐	☐	☐	☐
2	The quality of interaction between tutor and trainee was good.	☐	☐	☐	☐
3	The tutor could adequately follow the trainee during console tasks.	☐	☐	☐	☐
4	The trainee appeared to receive instructions promptly and clearly.	☐	☐	☐	☐
5	Tutor takeover, when required, occurred at the appropriate moment.	☐	☐	☐	☐
6	This format could be replicated safely on real patients.	☐	☐	☐	☐
7	Overall, the session was well organized.	☐	☐	☐	☐

Section B. Priority ranking (1 = most important; 5 = least important)

Item	Rank (1-5)
Clarity of tutor-to-trainee communication	_____
Adequacy of tutor takeover timing	_____
Atmosphere for learning	_____
Overall interaction quality	_____
Practical feasibility of the session set-up	_____

Section C. Overall satisfaction

The overall satisfaction of my experience with this training session was:

Very low ☐ Low ☐ High ☐ Very high ☐

Supplementary Table S1 - Shared tutor/trainee perception items

Item-level frequencies for the shared tutor/trainee perception items across the 30 paired tutor-trainee sessions.

Item pair (N = 30 paired sessions)	Tutor distribution	Trainee distribution	Weighted kappa	p-value
Session made teaching/training easy	SD 0; D 3; A 15; SA 12	SD 0; D 3; A 17; SA 10	0.47	0.004
Tutor could follow trainee / trainee felt followed	SD 0; D 3; A 16; SA 11	SD 0; D 4; A 17; SA 9	0.42	0.011
Quality of interaction was good	SD 0; D 2; A 15; SA 13	SD 0; D 3; A 15; SA 12	0.39	0.014
Promptness / clarity of instruction	SD 0; D 0; A 17; SA 13	SD 0; D 0; A 12; SA 18	0.18	0.170
Learning objectives achieved	SD 0; D 2; A 18; SA 10	SD 0; D 3; A 19; SA 8	0.13	0.290
Need for physical closeness	SD 16; D 10; A 3; SA 1	SD 5; D 9; A 10; SA 6	0.05	0.680

Supplementary Table S1. Shared tutor/trainee perception items by paired session.

Supplementary Table S2 - Specialist trainer/supervisor distributions by group

Distributions of selected specialist trainer/supervisor ratings in control and ReST sessions.

Trainer item	Control (n = 15)	ReST (n = 15)
Atmosphere for learning was adequate	SD 0; D 0; A 5; SA 10	SD 0; D 0; A 4; SA 11
Quality of interaction between tutor and trainee was good	SD 0; D 1; A 5; SA 9	SD 0; D 0; A 3; SA 12
Format could be replicated safely on real patients	SD 0; D 1; A 5; SA 9	SD 0; D 1; A 4; SA 10

Supplementary Table S2. Specialist trainer/supervisor distributions by group.

Supplementary Table S3 - Satisfaction counts by participant role and study group

Counts used for the satisfaction figure in the main manuscript and in Supplementary Figure S1.

Role / group	Very low	Low	High	Very high
Tutor - Control	0	0	8	7
Tutor - ReST	0	0	2	13
Specialist trainer/supervisor - Control	0	0	0	15
Specialist trainer/supervisor - ReST	0	0	2	13
Trainee - Control	0	0	6	9
Trainee - ReST	0	1	1	13

Supplementary Table S3. Satisfaction counts by participant role and study group.

Supplementary Figure S1 - Revised satisfaction figure

Grayscale-safe redraw of the post-session satisfaction figure using the counts reported in Supplementary Table S3.

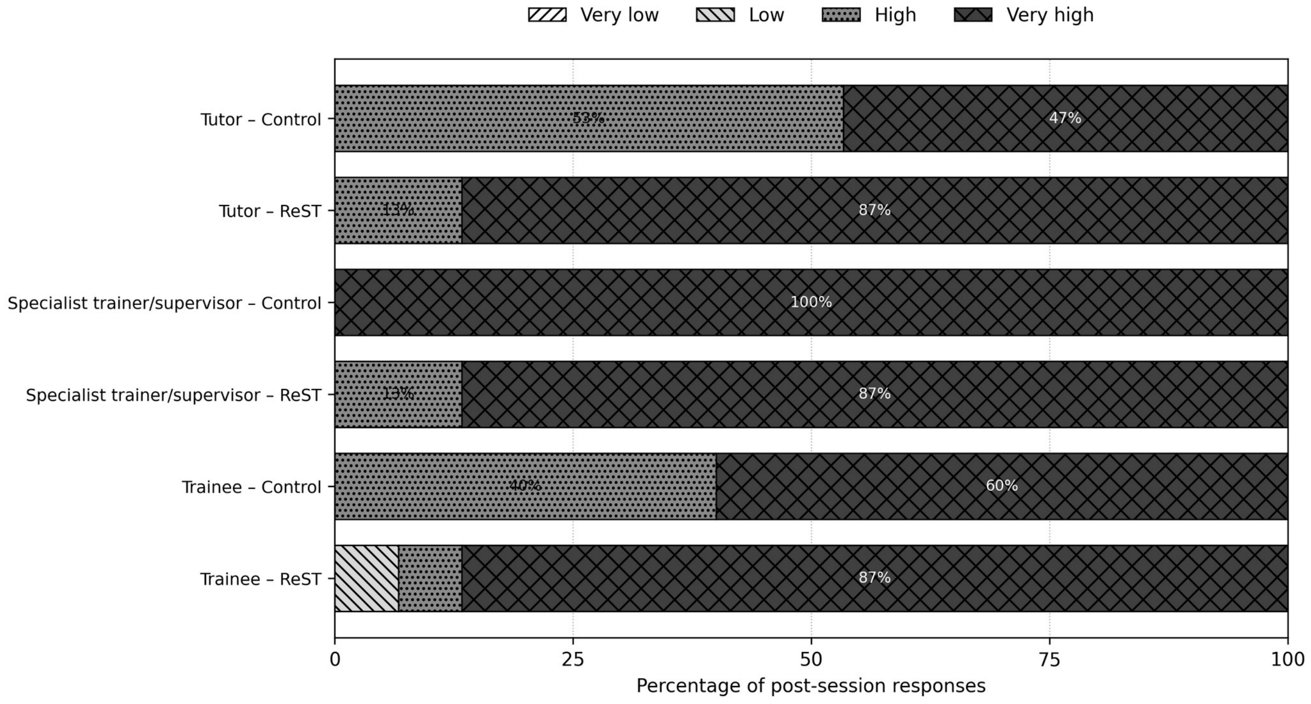

Supplementary Figure S1. Grayscale-safe redraw of the satisfaction figure.
